# Neural Implants Without Electronics: A Proof-of-Concept Study on a Human Skin Model

**DOI:** 10.1109/OJEMB.2020.2981254

**Published:** 2020-03-16

**Authors:** Patrick Kiele, David Braig, Jakob Weiß, Yara Baslan, Cristian Pasluosta, Thomas Stieglitz

**Affiliations:** ^1^ Laboratory of Biomedical MicrotechnologyDepartment of Microsystems Engineering–IMTEKUniversity of Freiburg 9174 79110 Freiburg Germany; ^2^ Department of Plastic and Hand Surgery, Medical Center-University of FreiburgFaculty of MedicineUniversity of Freiburg 9174 79106 Freiburg Germany; ^3^ Division of HandPlastic and Aesthetic SurgeryUniversity Hospital 80336 LMU Munich Germany; ^4^ Bernstein Center FreiburgUniversity of Freiburg 9174 79098 Freiburg Germany; ^5^ BrainLinks-BrainToolsUniversity of Freiburg 9174 79110 Freiburg Germany

**Keywords:** Capacitive coupling, electrode, neural implant, skin, telemetry

## Abstract

*Objective:* Chronic neural implants require energy and signal supply. The objective of this work was to evaluate a multichannel transcutaneous coupling approach in an *ex vivo* split-concept study, which minimizes the invasiveness of such an implant by externalizing the processing electronics. *Methods:* Herein, the experimental work focused on the transcutaneous energy and signal transmission. The performance was discussed with widely evaluated concepts of neural interfaces in the literature. *Results:* The performance of the transcutaneous coupling approach increased with higher channel count and higher electrode pitches. Electrical crosstalk among channels was present, but acceptable for the stimulation of peripheral nerves. *Conclusions:* Transcutaneous coupling with extracorporeal transmitting arrays and subcutaneous counterparts provide a promising alternative to the inductive concept particularly when a fully integration of the system in a prosthetic shaft is intended. The relocation of the electronics can potentially prevent pressure sores, improve accessibility for maintenance and increase lifetime of the implant.

## I. Introduction

The integration of somatosensory feedback in upper and lower limb prostheses has been shown to help patients in performing daily tasks such as object manipulation or walking on uneven ground [1]–[3]. Further, somatosensory feedback is essential to restore the sense of embodiment and agency, and may reduce phantom limb pain [4].

Recent studies demonstrated that electrical stimulation of peripheral nerves is an excellent alternative to stimulating the sensorimotor cortex, reducing the invasiveness of the system while producing an almost-natural and selective sense of touch [1]. Currently, different electrode designs exist to stimulate peripheral nerve pathways (e.g., TIME [5], TEENI [6], Cuff [7], LIFE [8], [9] and (Slanted [2]-SUEA) Utah electrode arrays-UEA [3]). However, energy and signal supply is limited to percutaneous cabling or inductive coupling.

Percutaneous cables penetrating the skin provide a relatively easy opportunity to interconnect implanted neural electrodes with an external stimulation device. A high risk of infection as well as skin irritation limit this approach to acute or short term treatments [10]–[13].

**Figure 1.**
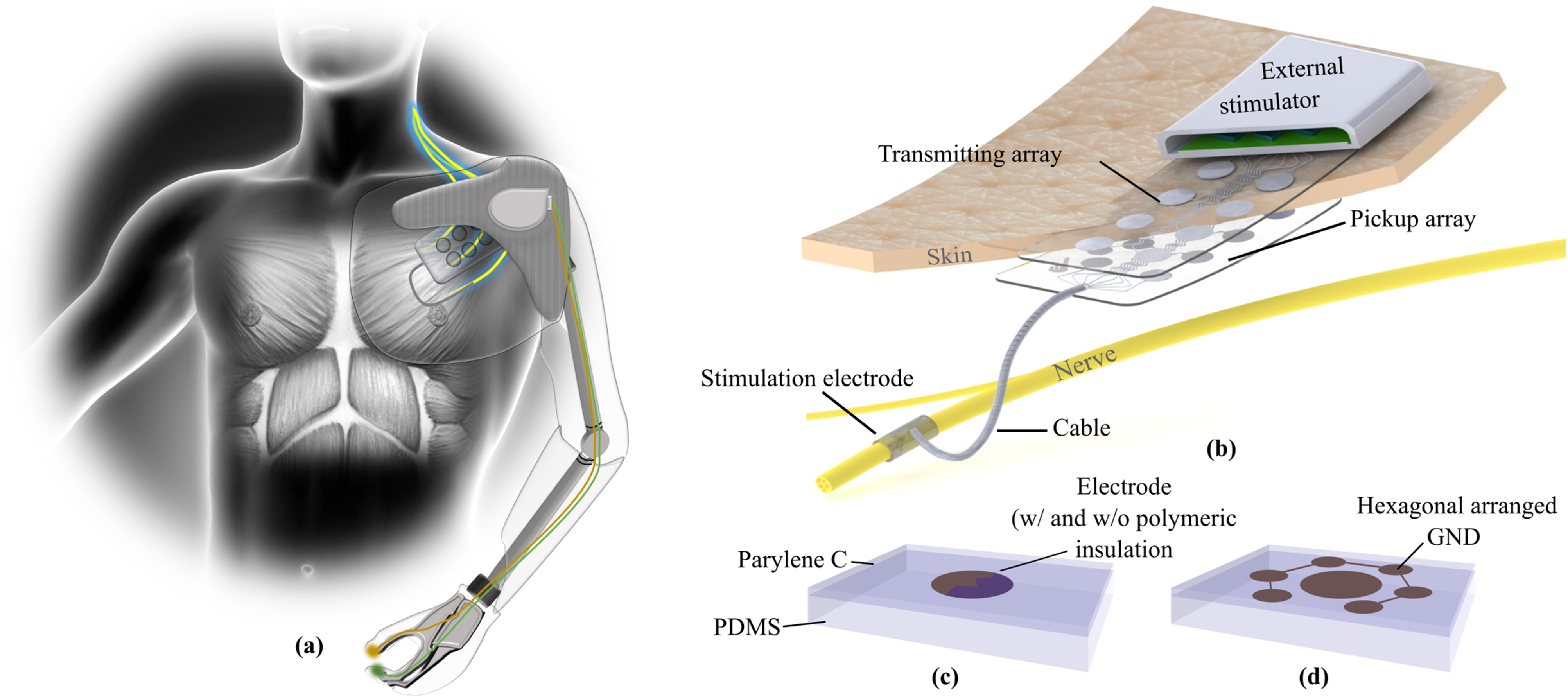
(a) Somatosensory feedback helps patients performing daily tasks. The tactile information from the prosthetic device needs to be processed and transferred transcutaneously in the body to stimulate peripheral nerves. (b) With a multichannel transcutaneous coupling approach the invasiveness of such a system can be minimized by providing the stimuli extracorporeal. The stimuli are picked-up at a subcutaneous electrode array before it is routed via implanted cables to the stimulation electrode. (c) In this study different electrode approaches with and without a polymeric insulation layer were evaluated. (d) The integration of hexagonally arranged ground electrodes in the transmitting array may help to minimize electrical crosstalk between adjacent coupling channels.

Telemetric communication through the skin presents a potential solution for these issues. Inductive coupling to provide energy and signals to implanted electronics is widely used for connecting neural implants (e.g., cochlear implants). However, these systems require costly and mostly bulky hermetic housings to preserve the electronics from failure due to body fluids, and more important to protect the surrounding tissue against toxic materials leaking out [14]. Thus, the implant becomes rather complex, providing a plurality of failure mechanisms that limit the lifetime of such systems. Here, we present a novel capacitive approach for wireless transcutaneous energy and signal supply to stimulate peripheral nerves. This approach eliminates the need for implanted electronics by providing the stimuli extracorporeally. The stimuli are transmitted capacitively through the skin via multiple electrode pairs forming plate capacitors with the skin as intermediate layer (Fig. 1b). The signal is then routed to the stimulation electrode. A similar concept was introduced by Gan and Prochazka with one single channel in their stimulus router system [15]. Contrary to the inductive approach, this implanted system consists only of thin and flexible structures that minimize invasiveness and allows full integration with the prosthetic socket, reducing the risk of pressure sores or permanent skin irritations (Fig. 1a).

Yet, the proposed concept for transcutaneous signal and energy supply poses significant design challenges. First, to provide proper somatosensory feedback multiple sensory pathways need to be stimulated simultaneously, making crosstalk during transcutaneous coupling an important issue. Second, the stimulation pulses are provided extra-corporeally, which implies that the signal shape and amplitude are deformed while propagating through the skin and are dependent of the skin conditions.

In this study, we separated the coupling part from the nerve interface and chose a translational approach by using human skin instead of rodents. We argue that an *ex vivo* human skin model delivers more realistic data for a future transfer of the parameters to a clinical application, additionally considering the 3R concept [16] of reduction, replacement and refinement of animal experiments in translational research [17]. We assessed three different capacitive multichannel coupling mechanisms using healthy explanted human skin samples. We evaluated the performance of the transcutaneous coupling as well as the crosstalk behavior. In particular, electrode arrays made of stainless steel base alloy (MP35N) or platinum iridium (PtIr) were used with either a direct metal-tissue contact or a polymeric insulation layer to block the resistive path (Fig. 1c). In some cases, ground electrodes were placed in a hexagonal arrangement around the electrodes to minimize the crosstalk behavior (Fig. 1d). We believe that the simplicity of this concept will increase longevity, robustness, and acceptance of invasive neural interfaces.

## II. Results

In this work, we focused on the evaluation of the coupling performance, since the stimulation paradigms of nerves has been already widely evaluated with different electrode designs [18]. The main question to be answered was whether the subcutaneous picked-up current was large enough to elicit nerve action.

### A. Coupling Behavior With Direct Metal Tissue Interfaces

**Figure 2.**
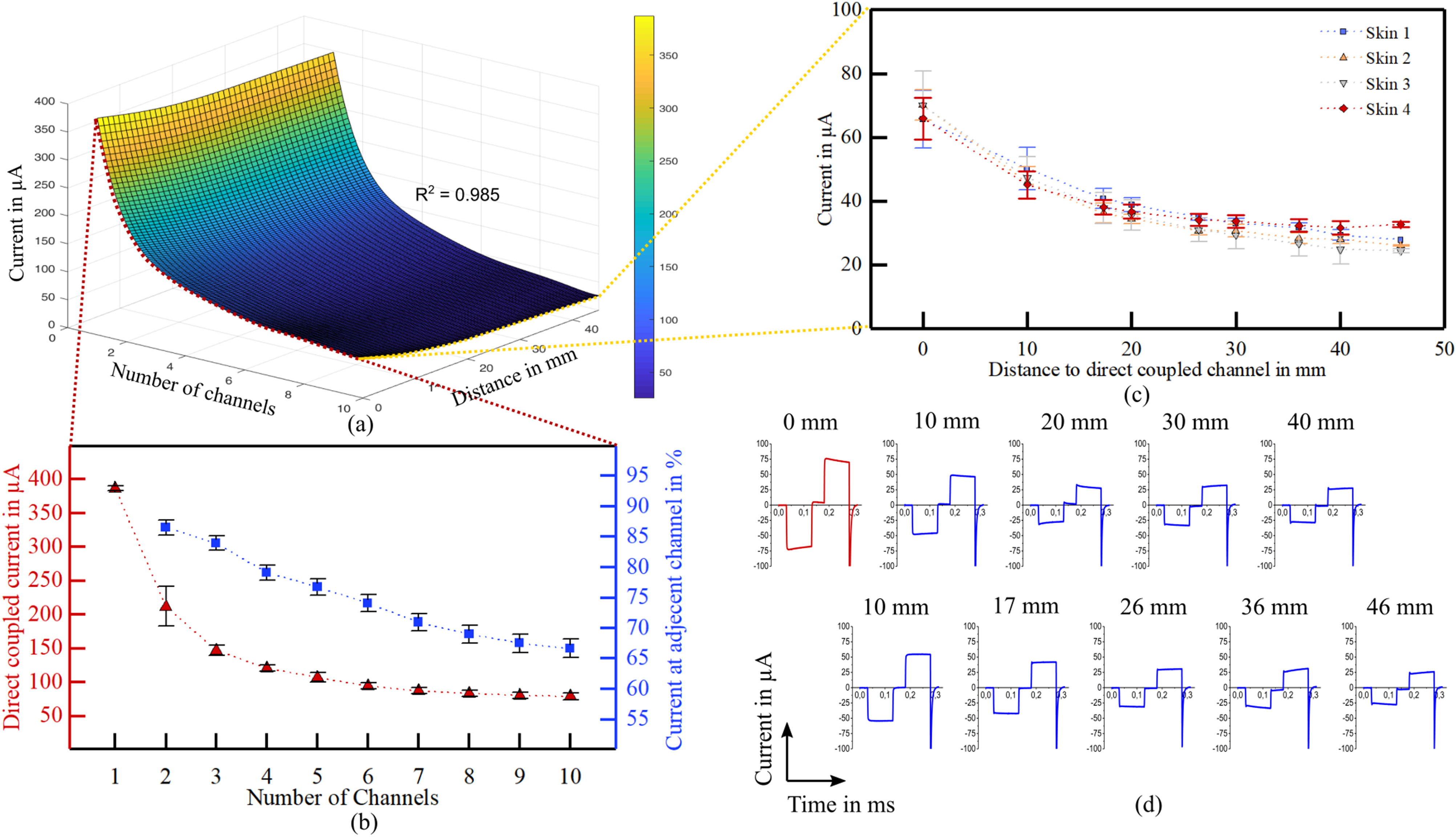
Current picked-up after transcutaneous coupling through explanted human skin samples. (a) A model of the current amplitudes as a function of the total number of transmitting channels and the lateral distance to the stimulation site was created including four different skin samples. (b) The maximum transferred current at the direct coupling path and the selectivity to an adjacent channel is influenced by the total number of channels implemented in the system. (c) With increasing lateral distance of the pickup electrode to the stimulation site the picked-up current decreases without significant differences in four different skin samples and thicknesses (t_skin 1_ = 4.5 mm, t_skin 2_ = 4.0 mm, t_skin 3_ = 3.5 mm, t_skin 4_ = 4.0 mm) (d) Current pulses after transcutaneous coupling at the pick-up array were slightly deformed compared to the biphasic square pulse at the extracorporeal electrode.

The evaluation of the electrical coupling of electrode arrays with a direct metal (MP35N) tissue contact was performed on four explanted human skin samples. We created a model of the picked-up current as a function of the total number of channels and the distance to the direct coupling electrode pair (i.e., distance = 0 mm) from a total of 1380 measurements (Fig. 2a).

The maximum coupled current at the pickup array showed a strong dependency to the number of channels, presenting the highest current with only one channel (386.57 ± 3.37 μA), and the lowest with ten channels (79.01 ± 4.74 μA). For small channel counts, the transmitted current was highly influenced by the electrode distance, whereby the influence decreased with higher channel counts (Fig. 2b). The current picked up at adjacent channels in relation to the direct coupled path (distance = 10 mm) was higher for channel count of two (86.5 ± 1.3%) and decreased with more channels to 66.6 ± 1.5% (Fig. 2b, blue). The current picked-up at the different electrode distances presented a maximum at the direct path (Fig. 2c, 0 mm distance, 63.93 ± 10.25 μA, 70.00 ± 10.68 μA, 70.00 ± 10.68 μA and 65.95 ± 6.57 μA for skin sample 1 to 4, respectively), and decreased with increasing distance (Fig. 2c, 22.46 ± 7.69 μA, 22.46 ± 7.69 μA, 24.43 ± 0.57 μA and 32.79 ± 0.82 μA for skin sample 1 to 4, respectively). Even though the skin samples were of varying thicknesses (t_skin 1_ = 4.5 mm, t_skin 2_ = 4.0 mm, t_skin 3_ = 3.5 mm, t_skin 4_ = 4.0 mm), no significant differences were observed in the coupling behavior (Fig. 2c, linear mixed model, F = 1.361, p = 0.255).

The pulse shapes measured at the pickup array had a square pulse characteristic, but were slightly deformed after the transcutaneous coupling (Fig. 2d). At short distances, there was a slight decrease in the amplitude of the plateau over time, while the trend was reversed with increasing distance. A short negative peak at the end of the pulse in each case occurred originating from forced charge balance of the stimulator system (Fig. 2d).

### B. The Influence of Electrode Pitch

The same electrode arrays were used to evaluate the influence of variating the electrode pitch. In this case, every second pick-up channel was electrically disconnected, resulting in an electrode pitch of approximately 20 mm. To compare the results with the 10 mm pitch, only measurements with five channels were included.

**Figure 3.**
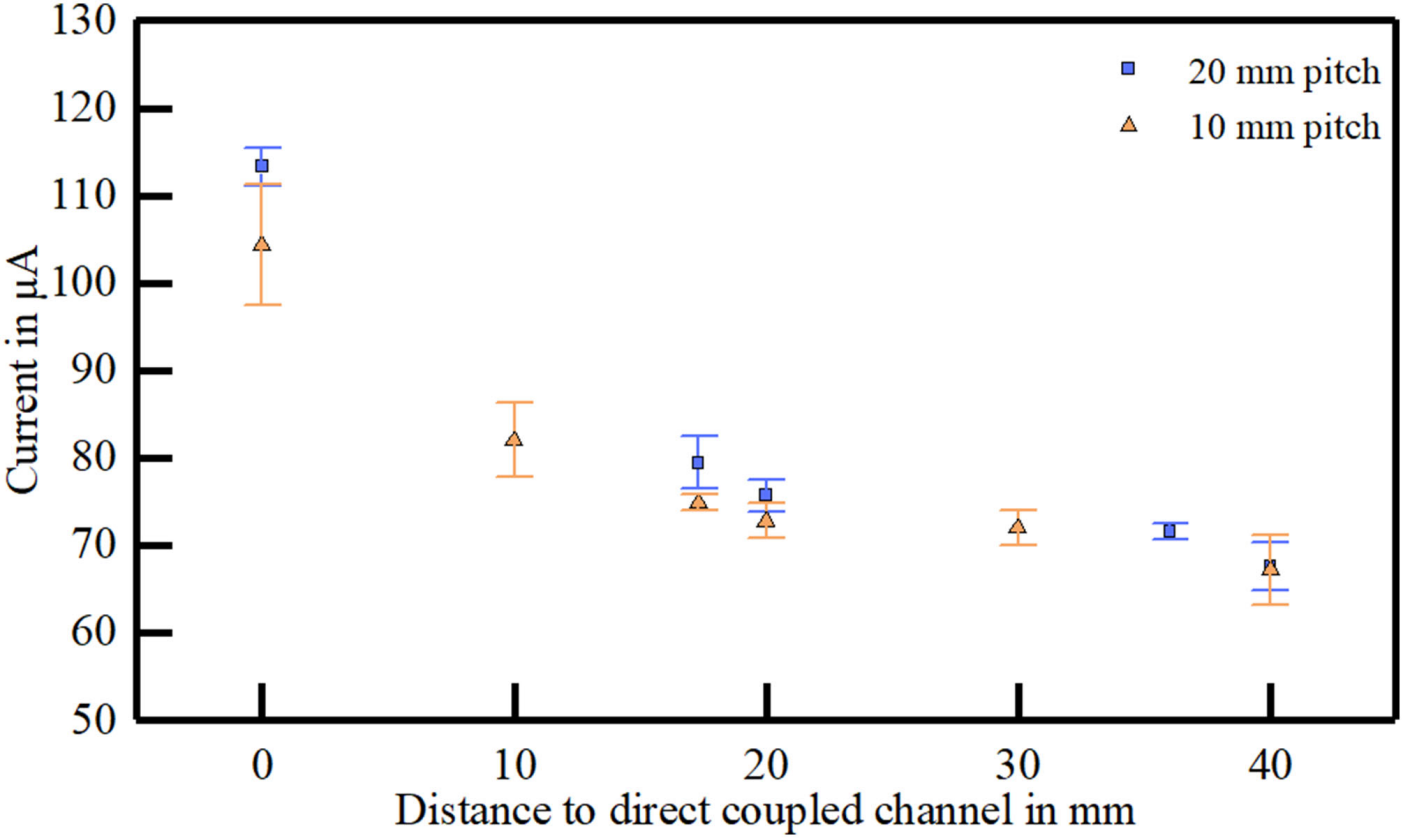
Current picked-up in five channel electrode arrays with electrode pitches of 10 mm and 20 mm. The measurements were performed on a single skin sample.

The coupling behavior of the different pitches presented a similar behavior, with a slightly higher current picked-up at the direct coupling path (+8.9 μA for the larger pitch, Fig. 3). Further, the amplitude difference decreased with increasing distance to a minimum of +0.3 μA (Fig. 3). Due to the small dataset (n = 2-4), no statistical analysis was performed.

### C. Integration of Extracorporeal Hexagonally Arranged Ground Electrodes

**Figure 4.**
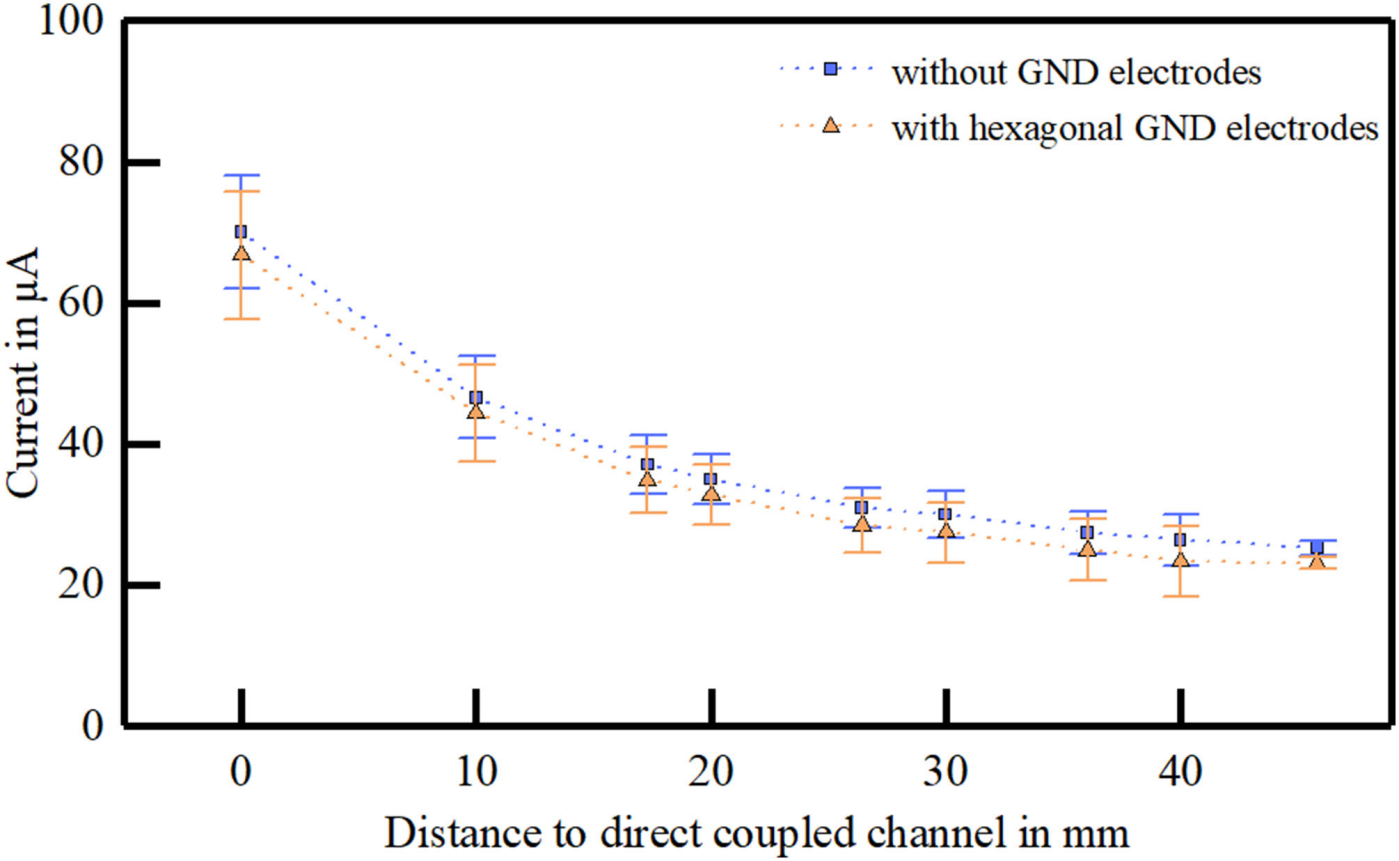
Influence of the integration of hexagonal ground electrodes on the transcutaneous coupled current in a ten channel system.

The influence of hexagonally arranged ground electrodes on the extracorporeal electrode array was evaluated on two skin samples. For this experiment, the metal part of the extracorporeal and the pick-up array were in direct contact with the skin tissue. The electrical coupling behavior indicated a significant alteration to the behavior without the implemented ground electrodes (F = 18.588, p < 0.001, Fig. 4). However, the overall behavior of the coupling characteristics was comparable in the observed range. When normalizing the coupling currents no significant change could be observed (F = 0.076, p = 0.783), supporting the comparable behavior, but indicating a general reduction of the amplitude of 2.50 ± 0.40 μA.

### D. Transcutaneous Coupling With a Blocked Resistive Path

**Figure 5.**
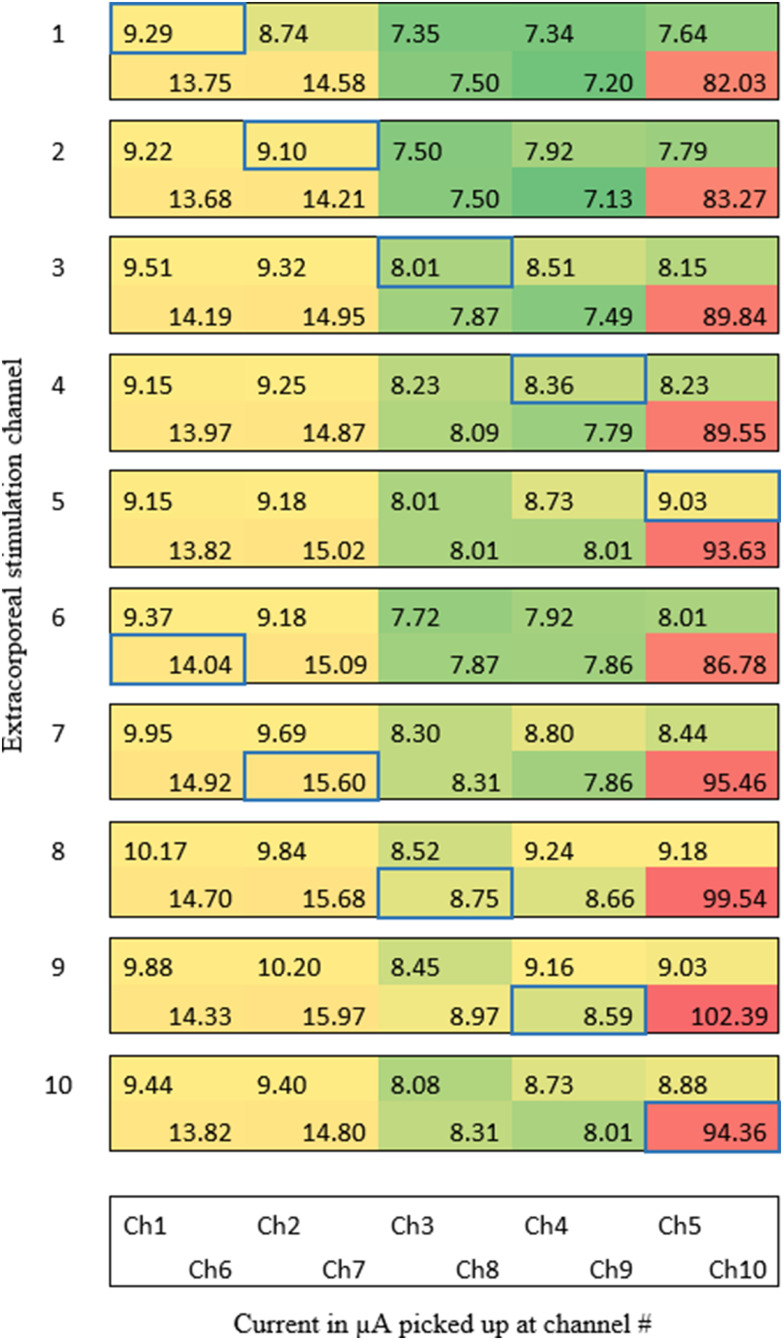
The maximal current at a ten channel pick-up array with polymeric insulation layer. The extracorporeal stimulation site is marked in blue.

We evaluated the transcutaneous coupling behavior using the electrode arrays with a polymeric insulation layer. The current at the pick-up array showed a characteristic coupling behavior in two skin samples. The picked-up current at one specific channel was almost constant, despite of the variation in the extracorporeal stimulation site (Fig. 5).

### E. Influence of the Electrode Material

The influence of different types of metals (MP35N, PtIr) was evaluated on two skin samples. An analogous coupling behavior was observed for the MP35N and PtIr electrode arrays with a polymeric insulation layer. The amplitude of the picked-up current was almost constant regardless of the extracorporeal stimulation site (Fig. 5).

**Figure 6.**
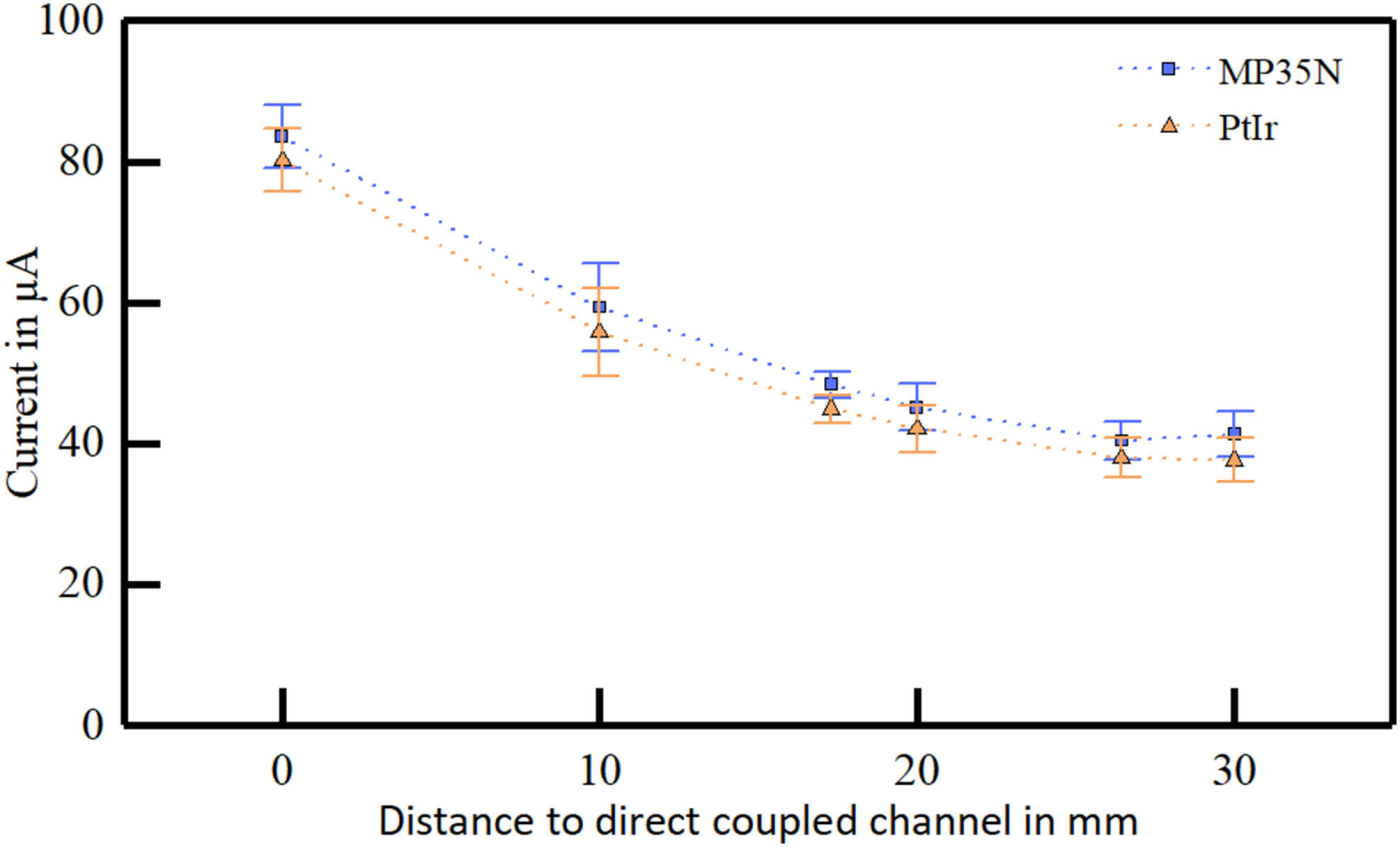
Influence of different electrode materials on the transcutaneous coupled current in a ten channel system in relation to the lateral distance to the extracorporeal stimulation site.

We evaluated the transcutaneous coupling behavior for the electrode arrays featuring metal-tissue interfaces on two different skin samples. The current distribution in the pick-up array significantly differed between the two metal types (F = 20.022, p < 0.001, Fig. 5). Nevertheless, the overall behavior of both metals was comparable, with an amplitude shift of 3.23 ± 0.04 μA along the whole range of distances (Fig. 6). These differences were not observed after normalizing the picked-up currents (F = 0.920, p = 0.339).

**Figure 7.**
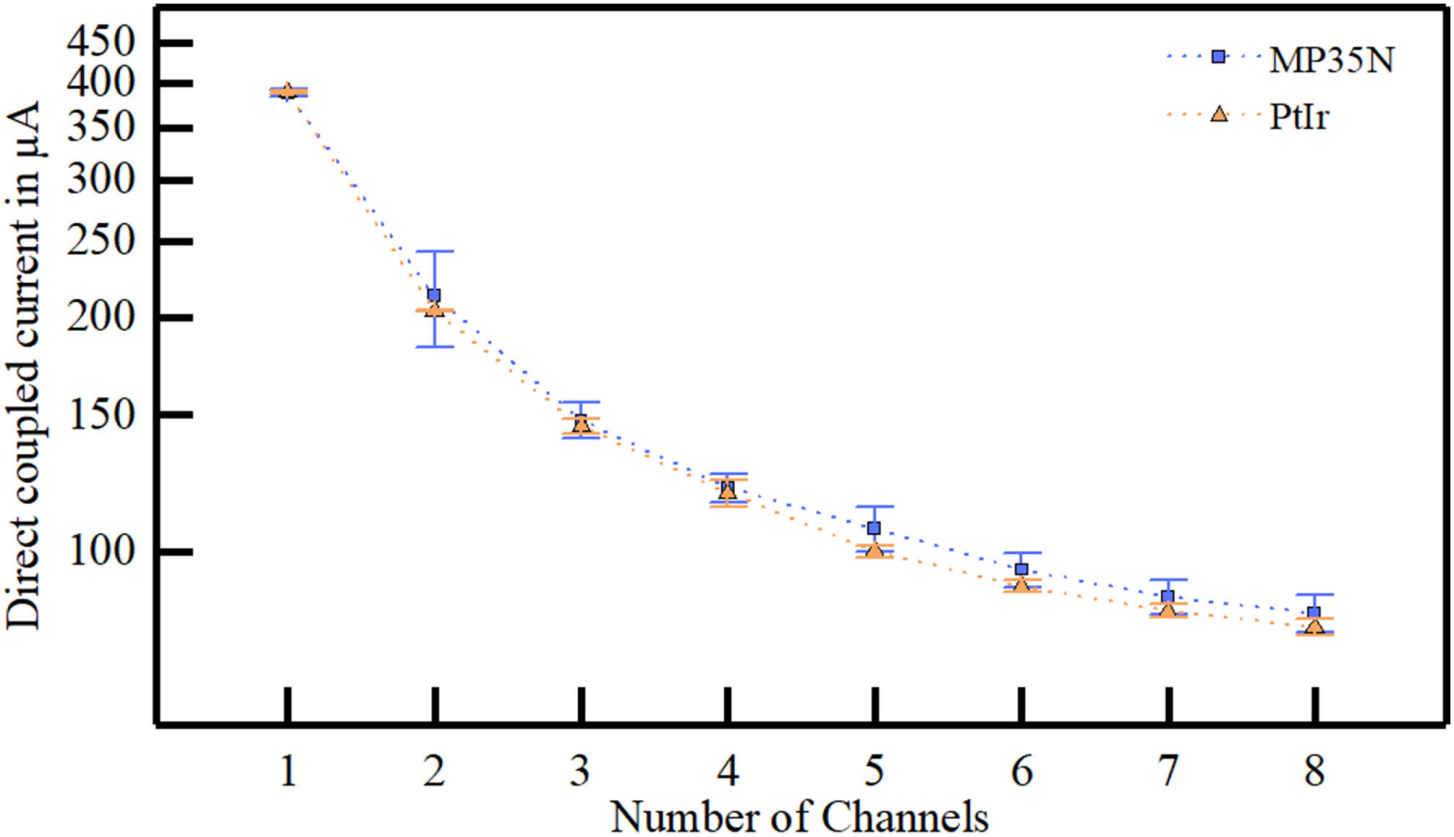
Influence of different electrode materials on the direct transcutaneous coupled current in relation to the number of electrodes.

We also compared the influence of the total number of channels on the direct coupling path. No significant difference between metal types was observed with absolute and normalized current amplitudes (F_absolute_ = 0.153, Fig. 7; F_normalized_ = 0.049, p = 0.826). The current amplitude was higher using electrodes made of MP35N (ΔI = 3.88 ± 0.04 μA).

## III. Discussion

To reduce the invasiveness of chronic neural implants we propose to relocate the processing electronics extra-corporeal and transmit the energy and signal for peripheral nerve stimulation transcutaneously via a multichannel coupling array. In this work, we evaluated different coupling strategies and designs on the crosstalk characteristics and coupling performance in an *ex vivo* study.

### A. Electrode Arrays With Polymeric Insulation

Human skin features excellent dielectric properties in the lower frequency range (ɛ_r,100 Hz_ = 1135.9, [19]) with a relatively low conductivity (σ_100 Hz_ = 0.0002 S/m, [19]). To minimize the crosstalk between adjacent electrodes, one may rely on those properties by integrating a thin insulation layer on top of the electrodes to block the resistive path in the skin, forming a plate capacitor. This study presents evidence that a purely capacitive transcutaneous coupling is possible. However, the amount of current picked-up was independent of the stimulation site, but it rather depended on the electrode itself. The peak in channel ten (Fig. 5) could be an indicator of an electrical pinhole in the insulation layer, which could be however not seen in electrochemical impedance characterizations.

### B. Electrode Arrays With Metal Tissue Interface

The electrical current distribution during transcutaneous coupling with electrode arrays featuring a direct metal-tissue interface was rather of resistive behavior within the skin with faradaic coupling mechanisms at the electrode interfaces. The maximum current amplitude picked-up at the direct coupling path, meaning the extracorporeal and subcutaneous electrode pair were aligned, was observed with one coupling channel. With increasing number of channels, the input current was distributed resulting in smaller amplitudes at the direct coupling path. With a total channel count of 10, roughly 20% of the input current was coupled through the direct path. However, the influence of the total amount of channels on the direct coupled path became smaller for greater channel counts (>7), while the selectivity between adjacent channels increased constantly.

With multiple coupling channels, the current distribution can be divided in two regions. With distances in the range of two times the skin thickness (taking the lateral dimensions of the electrode into account), the coupling was dominated by the vertical resistive path, while for bigger distances the lateral resistive path leads to a linear decrease of the picked-up current.

However, for the purpose of this concept – delivering stimuli for the stimulation of peripheral nerves – two main questions arise: 1) Is the crosstalk during transcutaneous coupling acceptable for a selective multichannel stimulation? And 2) Is the charged delivered sufficient to actually stimulate nerve fibers?

#### 1) Crosstalk Behavior

The acceptable crosstalk of the coupling system is given by the threshold for exciting an action potential in the nerve, which is described in the strength duration curve [20]. Studies on sensorimotor stimulation of the sciatic nerve showed that this curve is highly specific [21], meaning that some degree of crosstalk is still acceptable. In our study, for a ten channel array with an electrode pitch of 10 mm, roughly 66% of the direct coupled current is delivered at the adjacent electrode, which might be sufficient for a selective stimulation. Since the behavior of a higher pitch is analogous, the amount of current at the adjacent channel can be further decreased.

#### 2) Amplitude

The stimulation threshold of nerve fibers is influenced by the thickness of the nerve fiber and the distance to the stimulation electrode [22]. Further, the required amplitude depends of the stimulation electrode design and implantation site (e.g., intrafascicular or extrafascicular) [23]. The applied amplitude reported in the literature ranges from 10–12 μA (contact between hand and object, tingle, pressure and movement) [24], 17–70 μA (touch, joint movement and position stimulation) [25], 1–100 μA (tingle, pressure, vibration, hair and air brush cold) [3] to amplitudes up to 2 mA (touch) [26]. Additionally, the required amplitude varies with the stimulation strategy (monopolar or bipolar) and the placement of the ground electrode. In this work, we target monopolar stimulation with a surface ground electrode where typically higher current amplitudes for peripheral nerve stimulations have been reported [7].

The achieved transcutaneous coupled current reported in this paper was 67.50 ± 8.55 μA for a ten channel array, indicating that this might be sufficient for some applications but might be too small for a monopolar stimulation in the presented arrangement [23]. In this study, the applied current peak was limited by the compliance voltage (10 V) of the stimulator. To increase the coupled currents, we propose to use a current driven stimulator with a higher compliance voltage in the safety limit for extracorporeal stimulation [21].

### C. The Influence of Extracorporeal Hexagonally Arranged Ground Electrodes

The concept of the hexagonally arranged ground electrodes was adapted from stimulation strategies in retinal implants [27]. We expected a decrease of the crosstalk behavior, since the ground electrodes potentially lead lateral currents away. In fact, we could not detect a significant improvement of the crosstalk behavior but only slightly smaller currents at the pick-up electrodes, which results from an additional current path in the system. Nevertheless, this approach should be considered for a long-term application providing a possible strategy to prevent surface currents on the skin due to sweat (not simulated in this paper) but probably limiting the depth in which excitation occurs [27]. Using current steering, this drawback might be overcome [28] at the cost of another monopolar electrode.

### D. Choice of the Metal

Considerations on metal in use should not be limited only on the coupling performance. Instead biocompatibility plays a major role for the extracorporeal and to a greater degree for the subcutaneous electrode array. In fact, the reported losses in the coupled current amplitude were rather small when using PtIr instead of MP35N. The material properties of PtIr offer more flexible subcutaneous electrode arrays, which plays an important role in chronic applications, specifically if the implantation site is prone to movements.

## IV. Conclusions

We successfully coupled stimulation pulses through human skin in an *ex vivo* proof-of-concept study using a multi-channel configuration with a transmitting electrode array placed on the skin and a subcutaneous counterpart picking up the signals. The presented approach represents a promising alternative to an inductive coupling for chronic neural implants, since the invasiveness is minimized. The energy transmission resulted in slightly deformed square pulses with an amplitude of 67.50 ± 8.55 μA in a ten channel configuration when applying a 400 μA stimulus. We propose to use a system with a minimum of seven channels, an electrode pitch of 20 mm and electrodes made of PtIr embedded in a PDMS–Parylene-C matrix. With the presented concept for energy and signal transmission implanted electronics can be avoided and common peripheral nerve interfaces can be coupled and driven with this multichannel concept. We believe that this is an important step towards a new generation of long-term stable, minimal invasive neural implants.

## V. Materials and Methods

### A. Stimulation Paradigms & Data Acquirement

**Figure 8.**
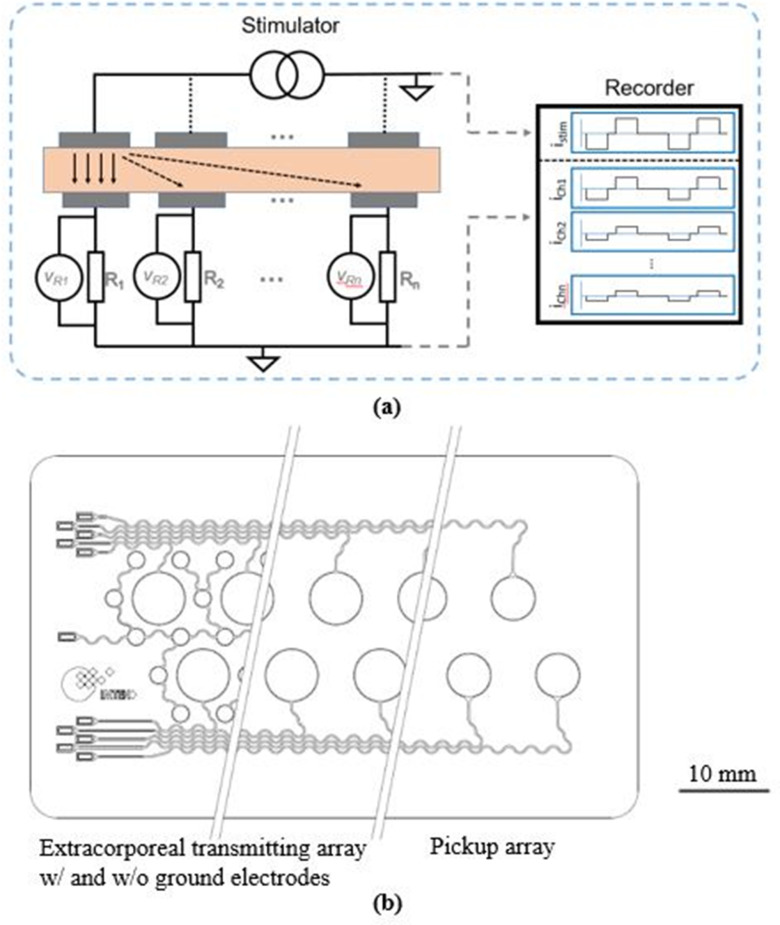
(a) Schematics of the measurement setup with a transmitting and a pick-up electrode array in a sandwich configuration with explanted human skin samples. (b) The transmitting electrodes had a diameter of 6 mm and in some cases hexagonally arranged ground electrodes (left/middle). The diameter of the pick-up electrodes was 5 mm to prevent misalignment errors.

The stimuli were driven by a current controlled stimulator (PlexStim, Plexon, Dallas, TX, USA) featuring a maximum current of 1 mA and a compliance voltage of up to 10 V. We applied a cathodic first charge balanced square pulse with an amplitude (*PA*) of 400 μA, a pulse width (*PW*) of 100 μs, an inter-pulse delay (*PD*) of 50 μs and a frequency (*f*) of 100 Hz at a single extracorporeal electrode placed on top of a skin sample. For electrodes featuring a polymeric insulation layer, the PA was 100 μA and the PW was 10 μs. The pick-up array was aligned and placed on the other side of the skin. Each pick-up electrode was connected via a resistor (*R =* 2.2 *kΩ*) for measuring the current to a common ground. A custom made multichannel recorder was developed in LabVIEW (V.2012, National Instruments, Austin, TX, USA) and NI DAQ (PXIe 6358 National Instruments, Austin, TX, USA) to collect all transmitted currents. Further specific electrodes could be switched off physically by interrupting the electric path to vary the number of channels used in the setup (Fig. 8a).

### B. Electrode Design and Fabrication Process

The electrodes were fabricated in a laser structuring process modified from [29]. Therefore, either MP35N or PtIr was embedded in a 180 μm thick PDMS (MED-1000, NuSil, Carpinteria, CA, USA) layer and covered with Parylene-C (10 μm, Special Coating Systems, Indianapolis, IN, USA). Parylene-C was removed on top of the electrodes sites for the fabrication of arrays with a metal-tissue interface.

Extracorporeal electrodes had a diameter of 6 mm, while the pick-up electrodes had a diameter of 5 mm to avoid misalignment errors. All electrode arrays had a pitch of 10 mm in a 2 × 5 configuration for the electrodes made of MP35N and 2 × 4 for PtIr respectively. The hexagonally arranged ground electrodes had a diameter of 2 mm with a pitch of 5 mm to the centered coupling electrode. These ground electrodes were only implemented in some extracorporeal arrangements (Fig. 8b).

### C. Skin Samples

Skin tissue was excess healthy human skin with subcutaneous tissue, taken from the abdomen of patients with tissue reduction surgery. Experiments were performed after removing fatty tissue within 4 h after tissue explantation to minimize alterations. Regular treatment with DPBS (ThermoFisher Scientific, Waltham, MA, USA) was performed to prevent dehydration. This study was approved by the ethical commission of the University Medical Center Freiburg (EK-Freiburg450/17). The design and performance of the study is in accordance with the Declaration of Helsinki. Signed informed consent was obtained from all participants.

### D. Statistics

A linear mixed model was used to test for fixed effects of distance, material, grounding and number of channels, using the measured current as the dependent variable. Electrode number and skin sample were considered random effects. Skin sample was considered a fixed effect in the case of analyzing the interaction between the skin sample effect and the distance effect. The significance was set at the α level of 0.05. All statistical analysis in this study were performed using R 3.5.1 (R Core Team, New Zealand).
